# Supplementary material for: A neural modeling approach to study mechanisms underlying the heterogeneity of visual spatial frequency sensitivity in schizophrenia
Source: bioRxiv. 2023 Oct 21:2023.10.18.563001. Preprint. [Version 1] doi: 10.1101/2023.10.18.563001 (PMC10614973; doi:10.1101/2023.10.18.563001)
Supplement: Supplement 1 [file NIHPP2023.10.18.563001v1-supplement-1.pdf]

bioRxiv preprint doi: https://doi.org/10.1101/2023.10.18.563001; this version posted October 21, 2023. The copyright holder for this preprint (which was not certified by peer review) is the author/funder, who has granted bioRxiv a license to display the preprint in perpetuity. It is made available under aCC-BY 4.0 International license.

## Supplementary Materials

**Supplementary Table 1. Parameter values per model run.** Parameters were varied across model trials. Across trials, A was held constant at 1, B was held constant at 10.1, C was held constant at 5, and a was held constant at 0.1. For a given trial, italicized values represent manipulated parameters, and non-italicized values represent values which were held constant. For each parameter, the color of the value matches the color of the respective curve that represents model outcome in Figures 3-9.

| Figure | $Amp_{ex}$ | $Amp_{inh}$ | $\sigma_{ex}$ | $\sigma_{inh}$ |
|---|---|---|---|---|
| 3 | 1 | 1 | *0.8*, *1.2* | 1.6 |
| 4 | *0.8*, *1.2* | 1 | 1 | 1.6 |
| 5A | *0.6* | 1 | *0.6* | 1.6 |
| 5C | *1.4* | 1 | *1.4* | 1.6 |
| 5E | *1.6* | 1 | *0.6* | 1.6 |
| 5G | *0.6* | 1 | *1.4* | 1.6 |
| 6 | 1 | 1 | 1 | *1.2*, *2* |
| 7 | 1 | *0.6*, *1.4* | 1 | 1.6 |
| 8A | 1 | *0.6* | 1 | *1.2* |
| 8C | 1 | *2* | 1 | *2* |
| 8E | 1 | *0.6* | 1 | *2* |
| 8G | 1 | *2* | 1 | *1.2* |
| 9A | 1 | 1 | *0.9*, *1.1* | *1.44*, *1.76* |
| 9C | *0.5*, *1.5* | *0.5*, *1.5* | 1 | 1.6 |

**Supplementary Table 2.** Summary of the model excitation and inhibition parameter search. Comparisons are made based on the model base parameters (control). Positive NDI value indicates increment relative to control, whereas negative NDI value indicates decrement relative to control.

| Modifications | Sensitivity at Low SF | Sensitivity at Mid SF | Sensitivity at High SF | Figure | cosSim and θ |
|---|---|---|---|---|---|
| ↓ $\sigma_{ex}$ | Increased<br>NDI = 0.0059<br>p = 3.9097e-9 | Increased<br>NDI = 0.0836<br>p = 1.1448e-13 | Increased<br>NDI = 0.2710<br>p = 6.0849e-15 | 3 (blue) | 0.9929<br>θ = 6.8284 |
| ↑ $\sigma_{ex}$ | Decreased | Decreased | Decreased | 3 (red) | 0.9945 |

bioRxiv preprint doi: https://doi.org/10.1101/2023.10.18.563001; this version posted October 21, 2023. The copyright holder for this preprint (which was not certified by peer review) is the author/funder, who has granted bioRxiv a license to display the preprint in perpetuity. It is made available under aCC-BY 4.0 International license.

| | | | | | |
|---|---|---|---|---|---|
| | NDI = –0.0070<br>p = 2.6918e-9 | NDI = –0.1039<br>p = 7.5829e-16 | NDI = –0.3045<br>p = 1.6987e-9 | | θ = 6.0091 |
| ↑ $Amp_{ex}$ | Increased<br>NDI = 0.1662<br>p = 7.8640e-160 | Increased<br>NDI = 0.1288<br>p = 1.0093e-20 | Increased<br>NDI = 0.0986<br>p = 8.0360e-7 | 4 (red) | 0.9993<br>θ = 2.1131 |
| ↓ $Amp_{ex}$ | Decreased<br>NDI = –0.2435<br>p = 1.6476e-161 | Decreased<br>NDI = –0.1717<br>p = 6.7283e-21 | Decreased<br>NDI = –0.1227<br>p = 7.9025e-7 | 4 (blue) | 0.9970<br>θ = 4.4131 |
| ↓ $\sigma_{ex}$,<br>↓ $Amp_{ex}$ | Decreased<br>NDI = –0.6193<br>p = 1.1876e-133 | Decreased<br>NDI = –0.2342<br>p = 9.9925e-11 | Increased<br>NDI = 0.2483<br>p = 9.5988e-12 | 5 (blue) | 0.7932<br>θ = 37.5104 |
| ↑ $\sigma_{ex}$<br>↑ $Amp_{ex}$ | Increased<br>NDI = 0.2760<br>p = 2.3574e-98 | No significant change*<br>NDI = 0.0327<br>p = 0.1642 | Decreased<br>NDI = –0.4378<br>p = 1.8774e-9 | 5 (red) | 0.9763<br>θ = 12.5071 |
| ↑ $\sigma_{ex}$<br>↓ $Amp_{ex}$ | Decreased<br>NDI = –0.6593<br>p = 1.1428e-248 | Decreased<br>NDI = –0.6726<br>p = 2.5923e-32 | Decreased<br>NDI = –0.7920<br>p = 1.3689e-7 | 5 (cyan) | 0.9851<br>θ = 9.8925 |
| ↓ $\sigma_{ex}$<br>↑ $Amp_{ex}$ | Increased<br>NDI = 0.2949<br>p = 1.0807e-167 | Increased<br>NDI = 0.3525<br>p = 2.9644e-31 | Increased<br>NDI = 0.6015<br>p = 2.6993e-18 | 5 (green) | 0.9972<br>θ = 4.2567 |
| ↓ $\sigma_{inh}$ | Decreased<br>NDI = –0.0078<br>p = 1.6805e-9 | Decreased<br>NDI = –0.0930<br>p = 3.8630e-20 | Decreased<br>NDI = –0.1220<br>p = 3.2456e-06 | 6 (blue) | 0.9972<br>θ = 4.3175 |
| ↑ $\sigma_{inh}$ | Increased<br>NDI = 0.0078<br>p = 8.7600e-10 | Increased<br>NDI = 0.0631<br>p = 2.4475e-24 | Increased<br>NDI = 0.0317<br>p = 7.8256e-4 | 6 (red) | 0.9988<br>θ = 2.8309 |
| ↓ $Amp_{inh}$ | Increased<br>NDI = 0.1508<br>p = 3.3833e-126 | Increased<br>NDI = 0.0808<br>p = 1.4392e-12 | Increased<br>NDI = 0.0177<br>p = 7.7164e-4 | 7 (blue) | 0.9977<br>θ = 3.8652 |
| ↑ $Amp_{inh}$ | Decreased<br>NDI = –0.2219<br>p = 1.6086e-124 | Decreased<br>NDI = –0.0972<br>p = 1.7289e-12 | Decreased<br>NDI = –0.0184<br>p = 7.7252e-4 | 7 (red) | 0.9909<br>θ = 7.7142 |
| ↓ $\sigma_{inh}$<br>↓ $Amp_{inh}$ | Increased<br>NDI = 0.1475<br>p = 1.2082e-103 | Increased<br>NDI = 0.0358<br>p = 2.8095e-4 | Decreased<br>NDI = –0.0495<br>p = 2.7697e-7 | 8 (blue) | 0.9948<br>θ = 5.8385 |
| ↑ $\sigma_{inh}$<br>↑ $Amp_{inh}$ | Decreased<br>NDI = –0.8160<br>p = 1.8043e-95 | Decreased<br>NDI = –0.0944<br>p = 9.7492e-5 | Increased<br>NDI = 0.0201<br>p = 0.0012 | 8 (red) | 0.6081<br>θ = 52.544 |
| ↓ $\sigma_{inh}$ | Decreased | Decreased | Decreased | 8 (green) | 0.6166 |

bioRxiv preprint doi: https://doi.org/10.1101/2023.10.18.563001; this version posted October 21, 2023. The copyright holder for this preprint (which was not certified by peer review) is the author/funder, who has granted bioRxiv a license to display the preprint in perpetuity. It is made available under aCC-BY 4.0 International license.

| | | | | | |
|---|---|---|---|---|---|
| ↑ $Amp_{inh}$ | NDI = –0.9334<br>p = 2.2416e-189 | NDI = –0.6610<br>p = 2.6644e-22 | NDI = –0.3567<br>p = 1.0297e-5 | | θ = 51.9349 |
| ↑ $\sigma_{inh}$,<br>↓ $Amp_{inh}$ | Increased<br>NDI = 0.1541<br>p = 4.1412e-169 | Increased<br>NDI = 0.1136<br>p = 1.2753e-16 | Increased<br>NDI = 0.0363<br>p = 7.6472e-4 | 8 (cyan) | 0.9989<br>θ = 2.7390 |
| ↓ $\sigma_{ex}$<br>↓ $\sigma_{inh}$ | Decreased<br>NDI = -1.136e-4<br>p = 6.8145e-9 | Increased<br>NDI = 0.0139<br>p = 3.9674e-6 | Increased<br>NDI = 0.1196<br>p = 1.4066e-14 | 9 (blue) | 0.9991<br>θ = 2.4188 |
| ↑ $\sigma_{ex}$<br>↑ $\sigma_{inh}$ | Decreased<br>NDI = -1.375e-4<br>p = 0.0017 | Decreased<br>NDI = –0.0180<br>p = 2.4694e-7 | Decreased<br>NDI = –0.1269<br>p = 1.8983e-11 | 9 (red) | 0.9993<br>θ = 2.22053 |
| ↓ $Amp_{ex}$<br>↓ $Amp_{inh}$ | Decreased<br>NDI = –0.3462<br>p = 3.6232e-267 | Decreased<br>NDI = –0.3384<br>p = 1.7569e-29 | Decreased<br>NDI = –0.3339<br>p = 3.2359e-7 | 9 (cyan) | 0.9999<br>θ = 0.8400 |
| ↑ $Amp_{ex}$<br>↑ $Amp_{inh}$ | Increased<br>NDI = 0.2241<br>p = 2.2135e-226 | Increased<br>NDI = 0.2093<br>p = 3.0294e-27 | Increased<br>NDI = 0.2009<br>p = 3.4444e-7 | 9 (green) | 1.000<br>θ = 0.4878 |

* Though no significant change was observed according to the NDI, this is likely due to the fact that the spatial frequency sensitivity curve for ↑ $\sigma_{ex}$ and ↑ $Amp_{ex}$ intersects the base curve within the medium spatial frequency range nulling the paired t-test. Moreover, there are nuances across studies for the effect size sometimes with and without p-value indicating significance; see [9].
